# Return to work and cancer survivorship needs of breast cancer survivors: an observational prospective single-cohort study in Italy

**DOI:** 10.1038/s41598-026-45780-y

**Published:** 2026-03-27

**Authors:** Sara Paltrinieri, Luca Braglia, Francesca Bravi, Stefania Fugazzaro, Stefania Costi

**Affiliations:** 1 Research and EBP Unit, Health Professions Department, Azienda USL- IRCCS di Reggio Emilia, Via Giovanni Amendola n. 2, Reggio Emilia, 42122 Italy; 2 Public Health Sciences PhD Program, Department of Clinical Sciences and Community Health, Dipartimento di Eccellenza 2023-2027, University of Milan, Via della Commenda n. 21, Milan, 20122 Italy; 3 Clinical Trials Center, Infrastructure Research and Statistics, Azienda USL-IRCCS di Reggio Emilia, Via Giovanni Amendola n. 2, Reggio Emilia, 42122 Italy; 4 Department of Clinical Sciences and Community Health, Dipartimento di Eccellenza 2023-2027, University of Milan, Via della Commenda n. 21, Milan, 20122 Italy; 5 Physical Medicine and Rehabilitation Unit, Azienda USL-IRCCS di Reggio Emilia, Via Giovanni Amendola n. 2, Reggio Emilia, 42122 Italy; 6 Department of Surgery, Medicine, Dentistry and Morphological Sciences, University of Modena and Reggio Emilia, Via del Pozzo n. 71, Modena, 41124 Italy

**Keywords:** Breast neoplasms, Cancer survivors, Return to work, Sick leave, Work difficulty, Needs, Cancer, Health care, Medical research, Oncology, Risk factors

## Abstract

Because work participation is an important goal of individuals with breast cancer (BC), we aimed to investigate the factors that may influence return to work in employed individuals as well as cancer survivorship needs regardless of employment status. Adult individuals with BC were followed up for 12 months after surgery. The impact of patient characteristics on return to work and needs was explored using penalized generalized linear mixed models. Of the 111 participants, 85 were working age and employed. Having children (odds ratio (OR) 0.81), undergoing lymph node dissection (OR 0.66), having targeted therapy (OR 0.78), and having a physically demanding job (OR 0.64) and a more severe disability of the upper limbs (OR 0.98) seemed to be associated with reduced return to work, as did taking sick days between diagnosis and surgery (OR 0.74) and taking sick leave after surgery (OR 13.78). More severe anxiety (OR 0.66), reduced sleep quality (OR 0.40), and upper limb disability (OR 0.13) seemed associated with higher needs. These findings suggest that employment is shaped by key determinants, whose early identification may help mitigate adverse work outcomes and support the timely addressing of needs.

**Supplementary Information:**

The online version contains supplementary material available at 10.1038/s41598-026-45780-y.

## Introduction

Return to work is one of the important goals of the growing cohort of cancer survivors^1^, with 50% of new BC diagnoses occurring in working-age individuals in Italy^2^.

Work participation positively affects well-being and quality of life^3^; for cancer survivors, it facilitates their return to normalcy and social engagement, and it guarantees an income. Furthermore, work participation counteracts the decrease in productivity caused by cancer-related absenteeism, which carries costs for both companies and society^4^.

Although cancer survivors may reorganize their life priorities and place greater emphasis on their personal life in the first two years following a cancer diagnosis^5^, returning to work is still often perceived as an important step toward the restoration of normalcy^6^. Nevertheless, cancer survivors are 1.4 times more likely than healthy individuals to be unemployed^7^. Particularly for individuals with BC, sociodemographic and disease-related variables such as low education level, having no children, and having undergone a mastectomy and/or chemotherapy are associated with a higher likelihood of unemployment^8–10^. Notably, the duration of sick leave seems to be longer in the first year following diagnosis^11^, particularly for patients who have undergone chemotherapy or mastectomy plus radiotherapy^12^. Even though the side effects of treatments can persist for five years after BC diagnosis^12^, the likelihood of being employed is similar to that of the general population at the same time point^13^. Work-related variables affect the likelihood of work resumption. Psychologically and physically demanding jobs seem to hinder the return to work^14^. Among individuals with BC, difficulties may include limited arm mobility, fatigue, and psychological and cognitive issues^3,15^. Additionally, workplace relationships are crucial: supportive employers who support communication and accommodate work-related issues facilitate reintegration^16^, while negative attitudes hinder work participation^14,17^.

Thus, sociodemographic, disease-related, and work-related variables of an individual with BC may influence return to work at different time points throughout that individual’s cancer journey, increasing the risk of negative work outcomes.

In Italy, a few studies have investigated the return-to-work process: a population-based cross-sectional survey^18^, a longitudinal study on cancer survivors^19^, and a cross-sectional study on BC patients^20^. Although the latter found lower work ability among individuals living alone and those who had undergone a mastectomy, its cross-sectional design made it impossible to determine the causal relationship between potential determinants and employment outcomes or to identify any change that had occurred since diagnosis^20^. These gaps could be better filled through a prospective study.

Finally, treatment side effects impact the health status and quality of life of individuals with BC^21,22^, hindering their participation in other meaningful daily activities, not just employment^15,23^, which may not represent a priority at all stages of the disease trajectory. These non-work-related, unmet needs deserve greater attention and in-depth exploration. For example, patients may require support in physical, psychological, or social domains to improve health outcomes and regain normalcy. Addressing these broader needs also helps to contextualize the return-to-work process and underscores the complexity of cancer survivorship, thereby supporting a more patient-centered perspective. Nevertheless, cancer survivorship models do not always exhaustively address these needs, denoting a gap in the services provided^24^. In Italy, evidence on the needs of this population is still lacking.

Thus, the first aim of this study was to investigate return to work and the associated factors of unemployment, work difficulties, and long-term sick leave in employed individuals with BC; the second aim was to investigate the associated factors of cancer survivorship needs perceived by individuals with BC regardless of their employment status.

## Methods

### Study design and setting

This observational prospective single-cohort study, reported according to the STROBE guidelines (Online Resource 1, p. 1), was conducted at the local health authority Azienda USL–IRCCS di Reggio Emilia (Italy), which includes five provincial hospitals plus the main hospital and cancer research center, located in the city of Reggio Emilia; cancer treatment and research activities are concentrated at the main hospital. Part of the standard care of individuals with BC who have undergone sentinel or axillary lymph node removal, regardless of the hospital where breast surgery was performed, is an educational outpatient group session conducted by a physiotherapist, scheduled approximately two weeks after surgery. For this study, potential participants were approached at the end of the group session they attended; the study was explained, and the contact information of those who were interested was collected. The inclusion criteria were having had a diagnosis of BC, having attended the assigned outpatient group session, and being aged ≥ 18 years. Telephone contact took place approximately three days after the group session to answer any questions and to schedule the baseline appointment. Informed consent was obtained from all participants before data collection.

Recruitment was carried out between May 2022 and May 2023; follow-up lasted 12 months, until May 2024. During the 12-month follow-up period, participants were interviewed five times: T0 (baseline), T1 (one month after T0), T2 (three months after T0), T3 (six months after T0), T4 (12 months after T0).

The study was approved by the AVEN Ethics Committee (protocol number 2022/0035983) and was registered prospectively in ClinicalTrail.gov (ID NCT05309252, registration date 25/03/2022). All methods in the present study were performed in accordance with the relevant guidelines and regulations of the Declaration of Helsinki.

### Outcomes

For the first aim, the outcomes collected for working-aged and for employed participants were:

- return to work at baseline and at each follow-up time point. Return to work was defined as the occurrence of work resumption regardless of any accommodation in terms of work schedule or work tasks (i.e., individuals who returned to work with reduced work hours were classified as having returned to work). Accordingly, return to work was recorded as a dichotomous outcome (yes/no) at each follow-up time point.
- work-related difficulties at baseline and at each follow-up time point (for participants who had returned to work), which were categorized using the International Classification of Functioning, Disability and Health Core Set for Vocational Rehabilitation-Onco (Core Set-VR-Onco)^25^, which was recently adapted to a population of cancer survivors^26,27^.
- number of sick days at baseline and at each follow-up time point.

For the second aim, the outcome collected for participants regardless of age (working age or not) and employment status (employed or not) was:

- cancer survivorship needs perceived at baseline and at each follow-up time point. Needs were collected through the Supportive Care Needs Survey–Short Form (SCNS-SF34)^28,29^.

### Variables and assessment tools

At baseline, data were collected on sociodemographic (sex, age, education level, marital status, children, income range according to the tax bracket for total annual individual taxable income, residence, citizenship), work-related (type of worker, type of company, employment contract, working hours, night worker, shift worker, flexible work schedule, flexible work tasks, psychologically/physically demanding work, number of coworkers, work experience, disability certificate, Law 104 guaranteeing legal protection in the workplace according to Italian law in particular health conditions, i.e., paid monthly leave, work accommodations, return to work, work-related difficulties, and number of sick days), and disease-related information (type of tumor, staging, surgery, axillary lymph node dissection, chemotherapy, radiotherapy, hormone therapy, targeted therapy, rehabilitation), health-related quality of life, and treatment side effects (mood disturbances, sleep disorders, disability of the upper limb, social participation, and quality of life associated to specific symptoms and distress). At each follow-up time point, data was collected on work-related (return to work, work-related difficulties, number of sick days) and disease-related (type of treatment and rehabilitation) information, health-related quality of life, and treatment side effects.

For health-related quality of life, data were collected through the administration of the European Organization for Research and Treatment of Cancer (EORTC) QLQ-C30^30^. For potential treatment side effects, data were collected through the administration of the Hospital Anxiety and Depression Scale (HADS)^31^, the Pittsburgh Sleep Quality Index (PSQI)^32^, the Disability of the Arm, Shoulder and Hand (DASH) questionnaire^33^, the Reintegration to Normal Living Index (RNLI)^34,35^, the Functional Assessment of Chronic Illness Therapy-Fatigue Scale (FACIT-Fatigue)^36^, the Functional Evaluation of Cancer Therapy-Cognitive Function (FACT-Cog)^37^, and the FACIT Comprehensive Score for Financial Toxicity (FACIT-COST)^38^.

A detailed description of the variables and the assessment tools used is reported in Online Resource 2 (p. 3).

### Sample size

The convenience sample of approximately 110–120 individuals with BC was estimated considering the predefined eligibility criteria, local indicators of the Azienda USL–IRCCS di Reggio Emilia (229 patients who attended the outpatient group sessions in 2019), data available from the local Cancer Registry, and recruitment period. In a cross-sectional study conducted locally, the recruitment rate was 70%^18^. However, given the prospective nature of this study, we estimated a lower rate.

### Statistical analysis

A descriptive analysis was performed to illustrate sociodemographic, disease-related, and work-related characteristics of all individuals with BC at baseline. Among the employed participants, the rates of those who returned to work, the absolute number of those who reported work-related difficulties, and the number of participants taking sick leave were calculated at each follow-up time point. All sick days were considered regardless of the perceived relationship to cancer. If sick leave was continuous, all seven days of the week were counted. The needs values for the domains of the SCNS-SF34 were calculated at each follow-up time point.

Outcomes were studied individually as dependent variables of generalized (multivariate) linear models for repeated measures, assigning the role of independent variables to potentially associated factors. Particularly, determinants of return to work, work difficulties, (log)days of non-work-attendance, and total needs at each time point were explored using penalized (LASSO) generalized linear mixed models (families were respectively binomial, binomial, gaussian, gaussian) with penalization parameter λ obtained by minimization of 5-fold cross-validation prediction error and a random intercept associated to the patient considered^39^. LASSO penalized GLMM models were adopted based on the number of patients involved (especially for primary outcome, *n* = 85), the type of primary outcome (dichotomous), and the number and types of covariates of interest to be included (several categorical variables implying a higher number of coefficients to be estimated) so as to limit the risk of overfitting and typical issues with classical (non-penalized) estimation. Covariates included in the estimate were age, education level, marital status, children, income range, type of tumor, surgery, axillary lymph node dissection, adjuvant chemotherapy, radiotherapy, hormone therapy, targeted therapy, rehabilitation, follow-up time point, type of worker, working hours, shift worker, night worker, working during weekend, flexible work schedule, psychologically demanding work, physically demanding work, number of coworkers, work experience, work accommodations, disability certificate, sick leave from diagnosis to surgery, sick leave after surgery, mood disturbances, sleep disorders, disability of the upper limb, social participation, and quality of life associated to specific symptoms and distress. All the results come from multivariate penalized analyses, where all the variables above were considered and collected at the same time points for all the patients. For better interpretability of estimates, only the coefficients not shrunk down to values suggesting no difference (e.g., 1 for OR) are reported so as to keep the tables readable. Finally, standard inference tools like confidence intervals and p-values are not available for this kind of analysis, and, as with penalized estimation, no difference/asymmetry in how independent variables were considered is present; all variables above were analyzed in each model, and no variable was considered fixed/to be necessarily present in final estimation.

Missingness was rather limited, and no ad hoc procedure was considered, for simplicity’s sake. Statistical analysis was conducted using R (version 4.3.0) with package glmmLasso version 1.6.3^39^.

## Results

Of the 442 individuals with BC invited to participate, 119 verbally agreed to participate, but only 113 of them signed the informed consent. Two participants did not show up at the baseline appointment, despite having signed the informed consent. Overall, the number of participants at each time point were: 111 at baseline, 103 at T1, 99 at T2, 94 at T3, and 90 at T4 (Online Resource 3, p. 5, Flow diagram of participants). A total of 83 participants (73.5%) attended all follow-up appointments.

As regards dropouts, there were five at T1, five at T2, and three at T3. Missing data also occurred: three participants missed the T1 appointment but came to the subsequent appointments; two missed T2 but came to the subsequent appointments; three missed T3 but came to T4, and seven participants missed this final follow-up appointment.

Table 1 reports the sociodemographic and disease-related characteristics of the study cohort and the work-related information of the 85 participants (76.6%) who were employed at baseline. The mean age of all the participants was 53.5 years (± 8.9), and most had a high school diploma or a university degree (74.8%). Conservative surgery was the most frequent surgery modality (61.3%); slightly more than 15% of the participants had undergone axillary lymph node dissection. Among the participants who were working at baseline, most were employees (89.4%), in the private sector (65.9%), with a permanent work contract (84.7%). More than 50% reported a psychologically demanding job.

**Fig. 1:**
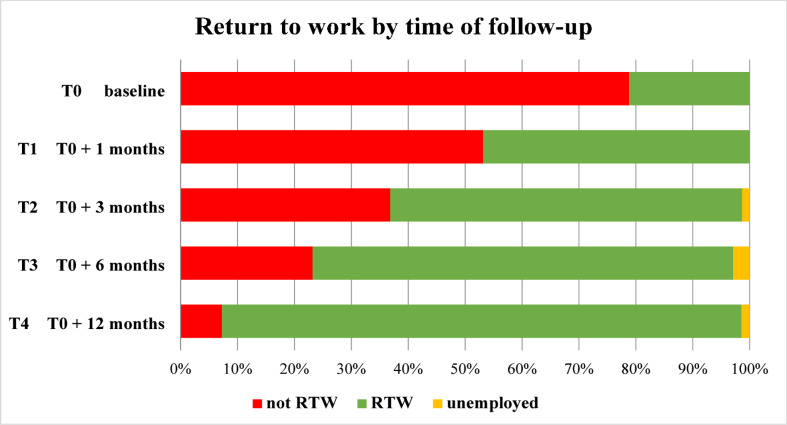
Rate of participants who returned to work.

Figure 1 describes the changes in the return to work of the 85 participants; these changes were recorded at baseline for all 85, for 79 at T1, 76 at T2, and 69 at T3 and at T4. The descriptive analysis suggested that, between baseline and T4, there was a progressive increase in the percentage of individuals with BC who had returned to work; at T4, 7.2% of participants had not yet returned to work. Online Resource 4 (p. 6) represents the changes in the return to work of the 85 participants categorized as employees or self-employed.

**Table 1:** Sociodemographic and Disease-Related Characteristics of 111 Participants at Baseline, and Work-Related Characteristics of 85 Participants who were Employed at Baseline.

		Total participants	Employed/Self-employed participants
		*n* (%)	*n* (%)
Sociodemographic characteristics			
Sex	Female	110 (99.1)	84 (98.8)
	Male	1 (0.9)	1 (1.2)
Age	Mean (SD)	53.5 (± 8.9)	50.9 (± 7.1)
	Median (range)	53 (28–76)	51.5 (28–66)
	< 50 years	37 (33.3)	38 (44.7)
	≥ 50 years	74 (66.7)	47 (55.3)
Education level	Middle school	27 (24.3)	17 (20.0)
	High school	59 (53.2)	45 (52.9)
	University degree	24 (21.6)	21 (24.7)
	Other	1 (0.9)	0 (0.0)
Marital status	Married	67 (60.4)	45 (52.9)
	Divorced	21 (18.9)	18 (21.2)
	Unmarried	17 (15.3)	14 (16.5)
	Cohabitant	5 (4.5)	5 (5.9)
	Widowed	1 (0.9)	1 (1.2)
Income	Up to €15,000	18 (16.2)	14 (16.5)
	€15,000–€28,000	49 (44.1)	37 (43.5)
	> €28,000–€50,000	38 (34.2)	27 (31.8)
	More than €50,000	5 (4.5)	4 (4.7)
	Unknown	1 (0.9)	1 (1.2)
Employment status	Employed/self-employed	85 (76.6)	85 (100.0)
	Unemployed	4 (3.6)	0 (0.0)
	Student/homemaker/retired	22 (19.8)	0 (0.0)
Children	0	24 (21.6)	17 (20.0)
	1	37 (33.3)	25 (29.4)
	≥ 2	50 (45.0)	41 (48.2)
Citizenship	Italian	104 (93.7)	78 (91.8)
	Italian and another one	4 (3.6)	3 (3.5)
	Not Italian	3 (2.7)	2 (2.4)
Cancer-related Characteristics			
Type of tumor	Ductal carcinoma	73 (65.8)	57 (67.1)
	Lobular carcinoma	13 (11.7)	9 (10.6)
	Other	5 (4.5)	4 (4.7)
	Unknown	20 (18.0)	15 (17.6)
Type of surgery	Conservative	68 (61.3)	48 (56.5)
	Mastectomy	42 (37.8)	36 (42.4)
	Axillary lymph node dissection	17 (15.3)	14 (16.5)
	Unknown	1 (0.9)	1 (1.2)
Treatment before surgery	Neoadjuvant chemotherapy	10 (9.0)	5 (5.9)
Work-related characteristics			
Work absence from diagnosis to surgery	No	/	40 (47.1)
	Yes	/	45 (52.9)
Type of worker	Employee	/	76 (89.4)
	Self-employed	/	9 (10.6)
Type of company	Private sector	/	56 (65.9)
	Public sector	/	29 (34.1)
Type of contract	Permanent	/	72 (84.7)
	Fixed term	/	3 (3.5)
	Other	/	10 (11.8)
Work schedule	Full-time	/	53 (62.4)
	Part-time	/	30 (35.3)
	Other	/	2 (2.4)
Number of workers in the company	Alone	/	5 (5.9)
	< 10	/	15 (17.6)
	10–49	/	18 (21.2)
	50–249	/	19 (22.4)
	≥ 250	/	28 (32.9)
Work experience (years)	< 1	/	1 (1.2)
	1–5	/	19 (22.4)
	5–10	/	6 (7.1)
	> 10	/	59 (69.4)
Shift worker	No	/	69 (81.2)
	Yes	/	16 (18.8)
Work on the weekend	No	/	53 (62.4)
	Yes	/	32 (37.6)
Work at night	No	/	78 (91.8)
	Yes	/	7 (8.2)
Flexible work schedule	No	/	46 (54.1)
	Yes	/	39 (45.9)
Flexible work tasks	No	/	46 (54.1)
	Yes	/	39 (45.9)
Psychologically demanding job	no	/	24 (28.2)
	Sometimes	/	16 (18.8)
	Yes	/	45 (52.9)
Physically demanding job	No	/	51 (60.0)
	Sometimes	/	7 (8.2)
	Yes	/	27 (31.8)

Online Resource 5 (p. 7) represents the work-related difficulties perceived during the return-to-work process. According to the descriptive analysis, the number of difficulties appeared to increase progressively from baseline to T4. The highest absolute numbers of problems in Body Functions (53 problems) and Environmental Factors (25 facilitators, 20 barriers) were reported at T3, while problems in Activity and Participation were more frequently reported at T4 (53 problems). At T3, the main problems concerned fatigue (category b455), sensation of pain (b280), lifting and carrying objects (d430), undertaking complex tasks (d220), and hand and arm use (d445). The main facilitators were receiving support from coworkers and employer (e325, e330); the main barrier was not receiving support from the employer (e330). At T4, fatigue (b455), sensation of pain (b280), and lifting and carrying objects (d430) were still reported as the main problems; complex relationships (category d720) were also reported among problems at this time point. Again, receiving support from coworkers and employer (e325, e330) were reported among facilitators at T4, while not receiving support from employer (e330) represented a barrier. A further barrier reported at T4 was prescribed drugs (e1101).

Online Resource 6 (p. 8) reports the number of participants employed at baseline and data regarding their sick leave calculated at baseline (from surgery) and for each subsequent time point. The number of participants who reported taking sick days decreased from baseline to the final follow-up appointment.

**Fig. 2:**
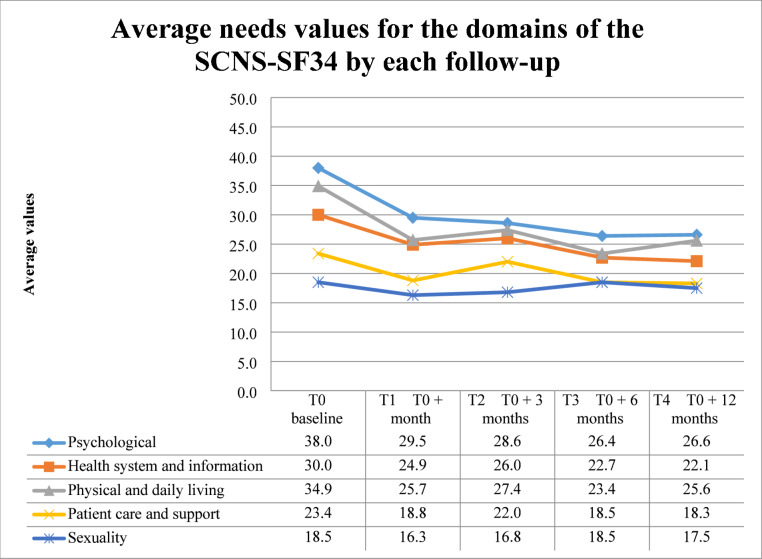
Average needs values for the domains of the SCNS-SF34 at each follow-up appointment.

Figure 2 shows the extent of needs for each domain of the SCNS-SF34 for all individuals with BC (111 participants at baseline). Overall, needs in all domains seemed to decrease between T0 and T1. At T2, needs in all domains increased, except for those in the psychological domain. At T3, needs in all domains decreased, except for those in the sexuality domain.

Table 2 reports the odds ratio (OR) for determinants of return to work, having difficulty, sick days, and total needs.

Being unmarried (OR 1.18) or divorced/widowed (OR 1.24) seemed to be associated with a higher likelihood of returning to work, as was a having higher income (OR 1.39). Having one child or more seemed to predict a lower rate of return to work (OR 0.81). Lobular carcinoma (OR 0.86), undergoing lymph node dissection (OR 0.66), and having targeted therapy (OR 0.78) seemed to be associated with less likelihood of returning to work. Self-employment (OR 1.42), having a flexible work schedule (OR 1.18), and having a disability certificate (OR 1.08) seemed to have higher return-to-work rates. Having taken sick days between diagnosis and surgery (OR 0.74), being a shift worker (OR 0.85), having a higher number of coworkers (OR 0.96 for 50–249 workers, OR 0.98 for ≥ 250 workers), and having a physically demanding job (OR 0.64) seemed to be associated with a lower return-to-work rate. Greater disability of the upper limbs (DASH OR 0.98) seemed negatively correlated to return to work. Follow-up time points seemed positively associated with return to work (OR 1.61–6.54).

A better quality of life (FACIT-Fatigue OR 0.93; FACT CogQoL OR 0.79; FACIT-COST 0.98) seemed positively correlated to a diminished likelihood of having work difficulty.

Having taken sick days between diagnosis and surgery appeared positively associated with taking sick leave after surgery (OR 13.78).

More severe symptoms (HADS-Anxiety OR 0.66 and HADS-Depression OR 0.78), sleep quality (PSQI OR 0.40), and disability of the upper limbs (DASH OR 0.13) seemed associated with higher total needs for both employed individuals with BC and all individuals with BC.

**Table 2:** Estimates for determinants of return to work, having difficulty, sick days, and total needs (regression model estimates and outcome reported in each gray sub-table header)

	Variables	OR Return to work
Sociodemographic characteristics		
Marital status	Married/cohabiting	ref.
	Divorced/widowed	1.24
	Unmarried	1.18
Number of children	Children	0.81
Income	Up to €15,000	ref.
	€15,000–€28,000	0.97
	> €28,000–€50,000	1.07
	More than €50,000	1.39
Cancer-related characteristics		
Type of tumor	Ductal carcinoma	ref.
	Lobular carcinoma	0.86
	Other	0.73
Type of surgery	Conservative surgery	ref.
	Mastectomy	0.99
Axillary lymph node dissection	No	ref.
	Yes	0.66
Adjuvant chemotherapy	No	ref.
	Yes	0.98
Radiotherapy	No	ref.
	Yes	0.97
Hormone therapy	No	ref.
	Yes	1.75
Targeted therapy	No	ref.
	Yes	0.78
Work-related characteristics		
Work absence from diagnosis to surgery	No	ref.
	Yes	0.74
Occupational status	Employed	ref.
	Self-employed	1.42
Shift worker	No	ref.
	Yes	0.85
Flexible work schedule	No	ref.
	Yes	1.18
Psychologically demanding job	No	ref.
	Sometimes	1.06
	Yes	0.99
Physically demanding job	No	ref.
	Sometimes	0.48
	Yes	0.64
Number of workers in the company	Alone	ref.
	< 10	1.05
	10–49	1.08
	50–249	0.96
	≥ 250	0.98
Disability certificate	No	ref.
	Recognition ongoing	0.79
	Yes	1.08
Potential treatment side effects	Social participation (RNLI)	1.02
	Depression (HADS)	1.04
	Sleep quality (PSQI)	1.01
	Cognitive function (FACT CogPCI)	0.99
	Financial toxicity (FACIT-COST)	1.02
	Upper limbs disability (DASH)	0.98
Follow-up timepoints	T0 baseline	ref.
	One month after T0	1.61
	Three months after T0	2.42
	Six months after T0	3.88
	12 months after T0	6.54

	Variables	OR Work difficulty
Potential treatment side effects	Fatigue (FACIT-Fatigue)	0.93
	Cognitive function (FACT CogQoL)	0.79
	Financial toxicity (FACIT-COST)	0.98

	Variables	Exp(beta) Sick leave
Potential treatment side effects	Work absence from diagnosis to surgery	13.78
	Social participation (RNLI)	0.99
	Upper limbs disability (DASH)	1.02

	Variables	Beta Needs employed individuals with BC
Potential treatment side effects	Social participation (RNLI)	− 0.18
	Anxiety (HADS)	0.70
	Depression (HADS)	0.75
	Sleep quality (PSQI)	0.44
	Fatigue (FACIT-Fatigue)	− 0.17
	Cognitive function (FACT CogPCI)	− 0.01
	Cognitive function (FACT CogQoL)	− 0.51
	Financial toxicity (FACIT-COST)	− 0.02
	Upper limbs disability (DASH)	0.14

	Variables	Beta Needs individuals with BC
Potential treatment side effects	Social participation (RNLI)	− 0.21
	Anxiety (HADS)	0.66
	Depression (HADS)	0.78
	Sleep quality (PSQI)	0.40
	Fatigue (FACIT-Fatigue)	− 0.17
	Cognitive function (FACT CogPCI)	− 0.01
	Cognitive function (FACT CogQoL)	− 0.54
	Financial toxicity (FACIT-COST)	− 0.03
	Upper limbs disability (DASH)	0.13

## Discussion

This study highlights associations between sociodemographic, work-related, and disease-related information, health-related quality of life, and treatment side effects with return to work, work difficulties, and sick leave among employed individuals with BC. Furthermore, it reports on the associations between potential predictors and the unmet cancer survivorship needs of adult individuals with BC.

Almost all employed participants in this study returned to work 12 months after surgery (91.3%), a rate higher than that reported in China (68.2%)^40^ and in France (42.2%)^41^. Among employed patients, factors such as having children, having undergone lymph node dissection, a diagnosis of lobular carcinoma, having received targeted therapy, having taken sick leave between diagnosis and surgery, being a shift worker, working in a large company, performing a physically demanding job, and experiencing greater upper limb disability seemed negatively associated with return to work. Having children was associated with a lower likelihood of returning to work, probably because family responsibilities and health concerns were prioritized over paid work^42^. Nevertheless, other studies did not confirm this association^8,9^. Lymph node dissection was associated with a reduced likelihood of returning to work, though the evidence remains inconsistent^9,43^. The influence of BC subtype remains largely unexplored, limiting comparability. Targeted therapy seemed to predict a lower likelihood of returning to work, although evidence from France suggests that this treatment modality does not affect work participation among individuals with localized BC^44^, although it does seem associated with sick leave three years after diagnosis^13^. Furthermore, three out of the four individuals with BC in our cohort who did not return to work underwent targeted therapy.

Shift work seemed negatively associated with the likelihood of returning to work. This finding is still poorly investigated in the literature^17^ despite its relevance to planning accommodations of work schedule. Employees in larger companies also seemed to be less prone to going back to work, possibly reflecting the extended sick leave policies in the public sector. Conversely, smaller companies may provide greater social supportive environments^45^ but fewer opportunities for workplace accommodations. The association between having a physically demanding job and a lower return-to-work rate was in line with previous findings from both cancer survivors in the same context^17^ and BC survivors^46^. This kind of work is generally more difficult to accommodate as it consists of predetermined work schedules, standardized methods of execution, a high degree of coordination with coworkers, and reduced decision latitude. Moreover, individuals with BC experience treatment-related side effects of the upper limbs, which further limits their ability to perform strenuous tasks. We found that greater upper limb disability was negatively associated with return to work, in line with previous findings retrieved one year after surgery^3^ and among patients who had undergone axillary lymph node dissection^47^. Follow-up time points were positively associated with return to work, with greater time since diagnosis increasing the likelihood of work resumption. This result, consistent with previous evidence^48^, should be interpreted alongside the descriptive finding that most participants reported work difficulties at T3 and at T4, when 73.9% and 91.3% of participants, respectively, had returned to work. While difficulties did not prevent work participation, they probably made the whole process more complicated as problems may arise afterward, possibly due to declining workplace social support or long-term treatment side effects. Hormone therapy seemed associated with work impairment but not with unemployment^49^. From a rehabilitation perspective, this finding could inform the timing of intervention^50^ to ease reintegration and prevent long-term adverse outcomes^51^.

Moreover, better quality of life associated with fatigue, cognition, and financial toxicity seemed to predict fewer work difficulties, consistently with the evidence showing that BC survivors who returned to work two years post-diagnosis reported less fatigue and cognitive impairment^52^.

Sick leave decreased between T0 and T4. In Finland, the highest percentage of sick leave days (68% during treatment) occurred among patients undergoing surgery, chemotherapy, and radiotherapy^53^. Similarly, chemotherapy and/or radiotherapy predicted delayed return to work in China^40^, whereas high income, not doing a physical job, and better physical functioning were associated with faster reintegration^40^. Our finding illustrates that taking sick days between diagnosis and surgery appeared negatively associated with return to work and positively associated with taking sick leave after surgery. Sick leave prior to surgery, rarely investigated in the literature, may reflect neoadjuvant treatment side effects or higher psychological distress at diagnosis^54^.

Cancer survivorship needs decreased between T0 and T1 but increased in most domains between T1 and T2; compared to T3, needs in psychological and physical and daily living domains increased. Moreover, psychological needs had the highest priority at each follow-up time point. Our recent systematic review showed that at one year or more after diagnosis, an increase in the trend of needs in all domains except for patient care and support needs was registered^55^. Greater anxiety, depression, sleep disturbances, and upper limb disability were linked to increased perceived needs. The odds ratios for employed individuals with BC and the total population were superimposable. Mental and physical health status and comorbidities seemed to predict unmet needs, consistently with a prior systematic review^55^.

One strength of this study is the low risk of recall bias due to the prospective nature of the study. However, it may have affected some factors (e.g., number of sick days), especially during the 6-month period between T3 and T4. Another possible strength is that there was only one rater. However, this could have led participants to providing answers that would make themselves be viewed more favorably in the eyes of the interviewer, introducing the social desirability bias^56^. Finally, the number of participants reached the amount hypothesized.

One limitation is that during the 12 months of follow-up, a dropout rate of 19% was recorded. Unfortunately, this may have been due to the demanding nature of the commitment, both in terms of the number of follow-up appointments and the number of administered questionnaires. To reduce the risk of drop out, participants were given the option of scheduling an appointment in person at the hospital closest to their home or else remotely. Another limitation concerns the eligibility criteria, as only individuals with BC who had undergone breast surgery with sentinel lymph node removal were included. In addition, the recruitment procedure may have introduced a selection bias. The study was presented following an outpatient group session all individuals with BC had been invited to attend after surgery, but participation was voluntary. This fact limits the generalizability of the results to individuals with BC with different clinical characteristics or those who did not attend the group session (e.g., those who had transport issues, more fragile individuals). From a research perspective, future studies should address this potential selection bias by adding alternative recruitment settings (e.g., first oncology consultation) in order to include a broader population. Moreover, although return to work is dynamic, in this study it was operationalized as a binary outcome assessed at multiple time points over 12 months post-surgery. Therefore, future studies should consider more nuanced outcome measures such as partial return to work or return to work with workplace accommodations and extend the follow-up period to better capture the evolution of return to work. Finally, future research should focus on formally testing the hypotheses generated by this exploratory study.

From clinical and rehabilitation perspectives, this prospective study allows early recognition of vulnerable individuals with BC, thereby facilitating the monitoring of their condition over time, supporting their return-to-work process, and addressing their needs through tailored solutions in line with the best evidence. The most effective interventions aimed at facilitating the reintegration of cancer survivors into the workplace appear to be multidisciplinary in nature^57^, relying on the coordinated involvement of different disciplines to address a broad range of work-related difficulties, for example, by including vocational counselling, patient education, and physical exercise. These interventions may be specifically targeted to individuals with BC^58,59^, whereas others are designed for cancer survivors regardless of their diagnosis^50,60^; the results of this prospective study could inform both types of intervention. These findings suggest that employment is shaped by key determinants, whose early identification in inpatient settings may help mitigate adverse outcomes. Particularly, our findings support the early identification of individuals with BC at higher risk of adverse work outcomes and those who could benefit more from a tailored monitoring of the work condition, which could start immediately after diagnosis or surgery. This underscores that healthcare professionals who interact with patients at risk must be adequately trained to identify and to proceed with a timely referral to the intervention. However, the timing of providing an intervention may vary; based on the descriptive data collected, interventions may be more appropriately concentrated several months following surgery or even after the actual return to work. Furthermore, the results of this study provide valuable information that can contribute to furthering the support offered to patients in the province of Reggio Emilia through the project UNAMANO^50^. As the intervention is intended for all cancer survivors, future research should verify the feasibility of the proposal in its current form and whether it adequately addresses the needs of individuals with BC.

Finally, survivorship needs extend beyond work-related aspects to encompass daily life. Because these needs evolve over the first year after surgery, they should be assessed according to the patient’s survivorship phase. This suggests that survivorship care interventions for individuals with BC should account for the variability in needs over time, which has been previously reported^55^, and patient-centered approaches to support the implementation of tailored survivorship care models should be adopted.

## Supplementary Information

Below is the link to the electronic supplementary material.

Supplementary Material 1

## Data Availability

The datasets generated and/or analyzed during the current study are available from the corresponding author on reasonable request.

## References

[1] Berger, I., Beck, L., Jones, J., MacEachen, E. & Kirsh, B. Exploring the needs of cancer survivors when returning to or staying in the workforce. *J. Occup. Rehabil*. **30**, 480–495 (2020). 32016649 10.1007/s10926-020-09877-z

[2] Global Cancer Observatory. Italy. (2022). https://gco.iarc.who.int/media/globocan/factsheets/populations/380-italy-fact-sheet.pdf

[3] Schmidt, M. E., Scherer, S., Wiskemann, J. & Steindorf, K. Return to work after breast cancer: The role of treatment-related side effects and potential impact on quality of life. *Eur J. Cancer Care (Engl*) **28**, e13051 (2019). 10.1111/ecc.13051 31033073

[4] Ferrier, C. et al. Absenteeism and indirect costs during the year following the diagnosis of an operable breast cancer: A prospective multicentric cohort study. *J. Gynecol. Obstet. Hum. Reprod.* **50**, 101871 (2021). 32673814 10.1016/j.jogoh.2020.101871

[5] Caumette, E. et al. Change in the value of work after breast cancer: evidence from a prospective cohort. *J. Cancer Surviv*. **17**, 694–705 (2023). 35267143 10.1007/s11764-022-01197-w

[6] Colombino, I. C. F., Sarri, A. J., Castro, I. Q., Paiva, C. E. & Da Costa Vieira, R. A. Factors associated with return to work in breast cancer survivors treated at the Public Cancer Hospital in Brazil. *Support Care Cancer*. **28**, 4445–4458 (2020). 31925532 10.1007/s00520-019-05164-7

[7] De Boer, A. G. E. M., Taskila, T., Ojajärvi, A., Van Dijk, F. J. H. & Verbeek, J. H. A. M. Cancer survivors and unemployment: A meta-analysis and meta-regression. *JAMA* **301**, 753 (2009). 19224752 10.1001/jama.2009.187

[8] Heuser, C. et al. Sociodemographic and disease-related determinants of return to work among women with breast cancer: A German longitudinal cohort study. *BMC Health Serv. Res.* **18**, 1000 (2018). 30594181 10.1186/s12913-018-3768-4 PMC6311058

[9] Wang, L. et al. Predictors of unemployment after breast cancer surgery: A systematic review and meta-analysis of observational studies. *J. Clin. Oncol.* **36**, 1868–1879 (2018). 29757686 10.1200/JCO.2017.77.3663 PMC6804906

[10] Tamminga, S. J. et al. Prognostic factors for return to work in breast cancer survivors. *Cochrane Database Syst. Rev.* (2025). (2025). 10.1002/14651858.CD015124.pub2 PMC12056893 40331515

[11] Kvillemo, P. K. et al. Sickness absence and disability pension among women with breast cancer: A population-based cohort study from Sweden. *BMC Public. Health*. **21**, 697 (2021). 33836707 10.1186/s12889-021-10703-1 PMC8033713

[12] Plym, A. et al. Impact of chemotherapy, radiotherapy, and endocrine therapy on sick leave in women with early-stage breast cancer during a 5-year period: A population-based cohort study. *Breast Cancer Res. Treat.* **182**, 699–707 (2020). 32506337 10.1007/s10549-020-05720-4 PMC7320921

[13] Monteiro, I. et al. Changes in employment status up to 5 years after breast cancer diagnosis: A prospective cohort study. *Breast* **48**, 38–44 (2019). 31493581 10.1016/j.breast.2019.07.007

[14] Paltrinieri, S. et al. Return to work in European Cancer survivors: A systematic review. *Support Care Cancer*. **26**, 2983–2994 (2018). 29845421 10.1007/s00520-018-4270-6

[15] Zomkowski, K. et al. Physical symptoms and working performance in female breast cancer survivors: A systematic review. *Disabil. Rehabil*. **40**, 1485–1493 (2018). 28325132 10.1080/09638288.2017.1300950

[16] Caron, M., Durand, M. J. & Tremblay, D. Perceptions of breast cancer survivors on the supporting practices of their supervisors in the return-to-work process: A qualitative descriptive study. *J. Occup. Rehabil*. **28**, 89–96 (2018). 28271399 10.1007/s10926-017-9698-x

[17] Paltrinieri, S. et al. Return to work of Italian cancer survivors: A focus on prognostic work-related factors. *Work* **71**, 681–691 (2022). 35253698 10.3233/WOR-210008

[18] Paltrinieri, S. et al. Factors influencing return to work of cancer survivors: A population-based study in Italy. *Support Care Cancer*. **28**, 701–712 (2020). 31129762 10.1007/s00520-019-04868-0

[19] Cavanna, L., Monfredo, M. & Citterio, C. La malattia cancro e la perdita del lavoro. Studio osservazionale prospettico su 416 pazienti oncologici con diagnosi di cancro e in attività lavorativa. 10.1701/3197.31748 31379371

[20] Musti, M. A. et al. Perceived work ability at return to work in women treated for breast cancer: A questionnaire-based study. *Med. Lav*. **109**, 407–419 (2018). 30556532 10.23749/mdl.v110i6.7241 PMC7682187

[21] Shapiro, C. L. & Recht, A. Side effects of adjuvant treatment of breast cancer. *N Engl. J. Med.* **344**, 1997–2008 (2001). 11430330 10.1056/NEJM200106283442607

[22] Ewertz, M. & Jensen, A. B. Late effects of breast cancer treatment and potentials for rehabilitation. *Acta Oncol.* **50**, 187–193 (2011). 21231780 10.3109/0284186X.2010.533190

[23] Loubani, K., Schreuer, N. & Kizony, R. Participation in daily activities among women 5 years after breast cancer. *Am. J. Occup. Ther.* **76**, 7604205050 (2022). 35767732 10.5014/ajot.2022.048736

[24] Carr, W. & Wolfe, S. Unmet needs as sociomedical indicators. *Int. J. Health Serv.* **6**, 417–430 (1976). 955751 10.2190/MCG0-UH8D-0AG8-VFNU

[25] World Health Organization. International classification of functioning disability and health. (2001). http://www.who.int/classification/icf/en/

[26] Paltrinieri, S. et al. Adaptation of the core set for vocational rehabilitation for cancer survivors: A qualitative consensus-based study. *J. Occup. Rehabil*. **32**, 718–730 (2022). 35334038 10.1007/s10926-022-10033-y PMC8949826

[27] Paltrinieri, S. et al. Validating the core set for vocational rehabilitation in a population of cancer survivors: A cross-sectional study. *J. Occup. Rehabil*. 10.1007/s10926-024-10252-5 (2024). 39663312 10.1007/s10926-024-10252-5 PMC12575594

[28] Zeneli, A. et al. Translation of supportive care needs survey short form 34 (SCNS-SF34) into Italian and cultural validation study. *Support Care Cancer*. **24**, 843–848 (2016). 26166001 10.1007/s00520-015-2852-0

[29] Zeneli, A. et al. Psychometric properties of the Italian version of the short-form supportive care needs survey questionnaire (SCNS-SF34-It): A multicenter validation study. *Nurs. Rep.* **14**, 303–316 (2024). 38391068 10.3390/nursrep14010023 PMC10885028

[30] Aaronson, N. K. et al. The European Organization for research and treatment of cancer QLQ-C30: A quality-of-life instrument for use in international clinical trials in oncology. *JNCI J. Natl. Cancer Inst.* **85**, 365–376 (1993). 8433390 10.1093/jnci/85.5.365

[31] Zigmond, A. S. & Snaith, R. P. The hospital anxiety and depression scale. *Acta Psychiatr Scand.* **67**, 361–370 (1983). 6880820 10.1111/j.1600-0447.1983.tb09716.x

[32] Curcio, G. et al. Validity of the Italian version of the Pittsburgh sleep quality index (PSQI). *Neurol. Sci.* **34**, 511–519 (2013). 22526760 10.1007/s10072-012-1085-y

[33] Padua, R. et al. Italian version of the disability of the arm, shoulder and hand (dash) questionnaire. Cross-cultural adaptation and validation. *J. Hand Surg.* **28**, 179–186 (2003). 10.1016/s0266-7681(02)00303-0 12631494

[34] Wood-Dauphinee, S. L., Opzoomer, M. A., Williams, J. I., Marchand, B. & Spitzer, W. O. Assessment of global function: The reintegration to normal living index. *Arch. Phys. Med. Rehabil*. **69**, 583–590 (1988). 3408328

[35] Wood-Dauphinee, S. & Williams, J. I. Reintegration to normal living as a proxy to quality of life. *J. Chronic Dis.* **40**, 491–499 (1987). 3597654 10.1016/0021-9681(87)90005-1

[36] Yellen, S. B., Cella, D. F., Webster, K., Blendowski, C. & Kaplan, E. Measuring fatigue and other anemia-related symptoms with the Functional Assessment of Cancer Therapy (FACT) measurement system. *J. Pain Symptom Manage.* **13**, 63–74 (1997). 9095563 10.1016/s0885-3924(96)00274-6

[37] Wagner, L., Sweet, J., Butt, Z., Lai, J. & Cella, D. Measuring patient self-reported cognitive function: Development of the functional assessment of cancer therapy–cognitive function instrument. *J. Supportive Oncol.* **7**, W32–W39 (2009).

[38] De Souza, J. A. et al. Measuring financial toxicity as a clinically relevant patient-reported outcome: The validation of the COmprehensive Score for financial Toxicity (COST). *Cancer* **123**, 476–484 (2017). 27716900 10.1002/cncr.30369 PMC5298039

[39] Groll, A. & Tutz, G. Variable selection for generalized linear mixed models by L 1-penalized estimation. *Stat. Comput.* **24**, 137–154 (2014).

[40] Ng, D. W. L. et al. Return to work, work productivity loss and activity impairment in Chinese breast cancer survivors 12-month post-surgery: A longitudinal study. *Front. Public. Health*. **12**, 1340920 (2024). 38463159 10.3389/fpubh.2024.1340920 PMC10920332

[41] Vayr, F. et al. Work adjustments and employment among breast cancer survivors: A French prospective study. *Support Care Cancer*. **28**, 185–192 (2020). 31001691 10.1007/s00520-019-04799-w

[42] Bøhn, S. K. H. et al. Work status changes and associated factors in a nationwide sample of Norwegian long-term breast cancer survivors. *J. Cancer Surviv*. **18**, 375–384 (2024). 35314959 10.1007/s11764-022-01202-2 PMC10960762

[43] Blinder, V., Patil, S., Eberle, C., Griggs, J. & Maly, R. C. Early predictors of not returning to work in low-income breast cancer survivors: A 5-year longitudinal study. *Breast Cancer Res. Treat.* **140**, 407–416 (2013). 23884596 10.1007/s10549-013-2625-8 PMC3826956

[44] Le Gall, G. et al. Impact of adjuvant trastuzumab treatment on fatigue, emotional status and quality of personal and work life of patients with localised breast cancer: Results of the ‘HER-ception’ study. *Support Care Cancer*. **31**, 38 (2023). 10.1007/s00520-022-07512-6 36525099

[45] De Ruiz, G. et al. Perceived discrimination at work: Examining social, health and work-related factors as determinants among breast cancer survivors – evidence from the prospective CANTO cohort. *J. Epidemiol. Community Health*. **76**, 918–924 (2022). 41877347 10.1136/jech-2021-218331

[46] Islam, T. et al. Factors associated with return to work of breast cancer survivors: A systematic review. *BMC Public. Health*. **14**, S8 (2014). 25437351 10.1186/1471-2458-14-S3-S8 PMC4251139

[47] Akezaki, Y. et al. Factors associated with return to work of breast cancer patients following axillary lymph node dissection. *Work* **70**, 271–277 (2021). 34511470 10.3233/WOR-213571

[48] Johnsson, A., Fornander, T., Rutqvist, L. E. & Olsson, M. Work status and life changes in the first year after breast cancer diagnosis. *Work Read. Mass.* **38**, 337–346 (2011). 10.3233/WOR-2011-1137 21508523

[49] Nakao, M. et al. The relationship between work-related outcomes and symptoms in early breast cancer survivors receiving adjuvant endocrine therapy. *Asia-Pac J. Oncol. Nurs.* **9**, 174–178 (2022). 35494090 10.1016/j.apjon.2022.01.003 PMC9052840

[50] Paltrinieri, S. et al. A social-healthcare pathway to facilitate return to work of cancer survivors in Italy: The UNAMANO project. *Work* **70**, 1243–1253 (2021). 34842210 10.3233/WOR-205249 PMC8764592

[51] de Ruiz, G. et al. Sustainable return to work among breast cancer survivors. *Cancer Med.* **12**, 19091–19101 (2023). 37602836 10.1002/cam4.6467 PMC10557874

[52] Lange, M. et al. Cognition and return to work status 2 years after breast cancer diagnosis. *JAMA Netw. Open.* **7**, e2427576 (2024). 39158915 10.1001/jamanetworkopen.2024.27576 PMC11333979

[53] Leskelä, R. L. et al. Predictive factors for prolonged sick leave in breast cancer patients treated with adjuvant therapies: A retrospective registry study. *Acta Oncol.* **62**, 1331–1337 (2023). 37699062 10.1080/0284186X.2023.2254483

[54] Fortin, J. et al. The mental health impacts of receiving a breast cancer diagnosis: A meta-analysis. *Br. J. Cancer*. **125**, 1582–1592 (2021). 34482373 10.1038/s41416-021-01542-3 PMC8608836

[55] Paltrinieri, S. et al. Needs of breast cancer survivors: A systematic review of quantitative data. *Crit. Rev. Oncol. Hematol.* **201**, 104432 (2024). 38955309 10.1016/j.critrevonc.2024.104432

[56] Furnham, A. Response bias, social desirability and dissimulation. *Personal Individ Differ.* **7**, 385–400 (1986).

[57] De Boer, A. G., Tamminga, S. J., Boschman, J. S. & Hoving, J. L. Non-medical interventions to enhance return to work for people with cancer. *Cochrane Database Syst. Rev.* (2024). 10.1002/14651858.CD007569.pub4 PMC10913845 38441440

[58] Algeo, N., Bennett, K. & Connolly, D. Rehabilitation interventions to support return to work for women with breast cancer: A systematic review and meta-analysis. *BMC Cancer*. **21**, 895 (2021). 34353286 10.1186/s12885-021-08613-x PMC8340442

[59] Sheppard, D. M. et al. Beyond Cancer’ Rehabilitation Program to support breast cancer survivors to return to health, wellness and work: Feasibility study outcomes. *Curr. Oncol.* **30**, 2249–2270 (2023). 36826135 10.3390/curroncol30020174 PMC9956005

[60] Zegers, A. D. et al. Supporting participation in paid work of cancer survivors and their partners in the Netherlands: Protocol of the SusTained Employability in cancer Patients and their partnerS (STEPS) multi-centre randomized controlled trial and cohort study. *BMC Public. Health*. **21**, 1844 (2021). 34641839 10.1186/s12889-021-11865-8 PMC8506084

