# Supplementary material for: Return to work and cancer survivorship needs of breast cancer survivors: an observational prospective single-cohort study in Italy
Source: Sci Rep. 2026 Mar 27;16:10827. doi: 10.1038/s41598-026-45780-y (PMC13039793; doi:10.1038/s41598-026-45780-y)
Supplement: Supplementary file 1 — Supplementary Material 1 [file 41598_2026_45780_MOESM1_ESM.pdf]

Online Resource 1. STROBE Statement—checklist of items that should be included in reports of observational studies.

	Item No	Recommendation	Page No
**Title and abstract**	1	(*a*) Indicate the study's design with a commonly used term in the title or the abstract	1
		(*b*) Provide in the abstract an informative and balanced summary of what was done and what was found	2
**Introduction**			
Background/rationale	2	Explain the scientific background and rationale for the investigation being reported	3-4
Objectives	3	State specific objectives, including any prespecified hypotheses	4
**Methods**			
Study design	4	Present key elements of study design early in the paper	4
Setting	5	Describe the setting, locations, and relevant dates, including periods of recruitment, exposure, follow-up, and data collection	4-5
Participants	6	(*a*) *Cohort study*—Give the eligibility criteria, and the sources and methods of selection of participants. Describe methods of follow-up<br>*Case-control study*—Give the eligibility criteria, and the sources and methods of case ascertainment and control selection. Give the rationale for the choice of cases and controls<br>*Cross-sectional study*—Give the eligibility criteria, and the sources and methods of selection of participants	4
		(*b*) *Cohort study*—For matched studies, give matching criteria and number of exposed and unexposed<br>*Case-control study*—For matched studies, give matching criteria and the number of controls per case	NA
Variables	7	Clearly define all outcomes, exposures, predictors, potential confounders, and effect modifiers. Give diagnostic criteria, if applicable	5-6
Data sources/ measurement	8*	For each variable of interest, give sources of data and details of methods of assessment (measurement). Describe comparability of assessment methods if there is more than one group	6
Bias	9	Describe any efforts to address potential sources of bias	6-7
Study size	10	Explain how the study size was arrived at	6
Quantitative variables	11	Explain how quantitative variables were handled in the analyses. If applicable, describe which groupings were chosen and why	6-7
Statistical methods	12	(*a*) Describe all statistical methods, including those used to control for confounding	6-7
		(*b*) Describe any methods used to examine subgroups and interactions	6-7
		(*c*) Explain how missing data were addressed	6-7
		(*d*) *Cohort study*—If applicable, explain how loss to follow-up was addressed<br>*Case-control study*—If applicable, explain how matching of cases and controls was addressed<br>*Cross-sectional study*—If applicable, describe analytical methods taking account of sampling strategy	6-7

		(*e*) Describe any sensitivity analyses	
**Results**			
Participants	13*	(a) Report numbers of individuals at each stage of study—eg numbers potentially eligible, examined for eligibility, confirmed eligible, included in the study, completing follow-up, and analysed	8
		(b) Give reasons for non-participation at each stage	8
		(c) Consider use of a flow diagram	Online resource 3
Descriptive data	14*	(a) Give characteristics of study participants (eg demographic, clinical, social) and information on exposures and potential confounders	8-12, Table 1
		(b) Indicate number of participants with missing data for each variable of interest	6-7
		(c) *Cohort study*—Summarise follow-up time (eg, average and total amount)	6-7
Outcome data	15*	*Cohort study*—Report numbers of outcome events or summary measures over time	6-7
		Case-control study—Report numbers in each exposure category, or summary measures of exposure	NA
		Cross-sectional study—Report numbers of outcome events or summary measures	NA
Main results	16	(*a*) Give unadjusted estimates and, if applicable, confounder-adjusted estimates and their precision (eg, 95% confidence interval). Make clear which confounders were adjusted for and why they were included	13, Table 2
		(*b*) Report category boundaries when continuous variables were categorized	13, Table 2
		(*c*) If relevant, consider translating estimates of relative risk into absolute risk for a meaningful time period	NA
Other analyses	17	Report other analyses done—eg analyses of subgroups and interactions, and sensitivity analyses	NA
**Discussion**			
Key results	18	Summarise key results with reference to study objectives	16-19
Limitations	19	Discuss limitations of the study, taking into account sources of potential bias or imprecision. Discuss both direction and magnitude of any potential bias	18-19
Interpretation	20	Give a cautious overall interpretation of results considering objectives, limitations, multiplicity of analyses, results from similar studies, and other relevant evidence	16-19
Generalisability	21	Discuss the generalisability (external validity) of the study results	16-19
**Other information**			
Funding	22	Give the source of funding and the role of the funders for the present study and, if applicable, for the original study on which the present article is based	25

Online Resource 2. Variables and assessment tools used to collect data on sociodemographic, work-related, and disease-related information, health-related quality of life, and treatment side effects.

Variables/assessment tool/checklist	Description
sociodemographic information	sex (male, female), age, education level (none, elementary school, middle school, high school, university degree, post-graduate degree, other), marital status (married, unmarried, divorced/separated, widowed, cohabiting), children (none, 1, ≥2), income range (up to €15,000, €15,000–€28,000, €28,000–€50,000, more than €50,000, unknown), residence, citizenship (Italian, Italian and another one, not Italian).
work-related information	type of worker (employed, self-employed), type of company (private, public, other), employment contract (permanent, fixed-term, other), work schedule (full-time, part-time, other), night worker (no, yes), shift worker (no, yes), flexible work schedule (no, yes), flexible work tasks (no, yes), psychologically demanding work (no, sometimes, yes), physically demanding work (no, sometimes, yes), number of coworkers (fewer than 10, from 10 to 49, from 50 to 249, more than 249, alone), work experience (less than 1 year, 1–5 years, 5–10 years, more than 10 years), disability certificate (no, yes, percentage), Law 104 (no, yes), work accommodations (no, yes), return to work (not yet returned to work, returned to work), work difficulties, number of sick days
disease-related information	type of tumor (ductal carcinoma, lobular carcinoma, other, unknown), staging (TNM), surgery (conservative surgery, mastectomy, I don't know), axillary lymph node dissection (no, yes), chemotherapy (neoadjuvant, adjuvant, months), radiotherapy (no, yes), hormone therapy (no, yes), targeted therapy (no, yes), rehabilitation (no, yes through outpatient individual physical therapy).
European Organization for Research and Treatment of Cancer (EORTC) QLQ-C30	The scale is a 30-item scale that measures health-related aspects that influence the quality of life of cancer patients. The QLQ-C30 is composed of three subscales: global health status (two items), functional scales (15 items), and symptom scale / individual items (13 items). Overall, it is possible to calculate 15 scores: one for the global health status, five for the functional scale, and nine overall for the symptom scale and individual items. Each item is assigned a score between 1 and 4 (4-point Likert scale), excluding items n. 29 and n. 30 (7-point Likert scale). All the subscales and single-item measures range in score from 0 to 100. A high scale score represents a higher response level. The higher the score for the global health status, the better the quality of life. The higher the score for the functional scale, the higher the level of functioning. Instead, the higher the score for symptom scale/items, the worse the symptoms.
Hospital Anxiety and Depression Scale (HADS)	The scale is a 14-item scale that assesses anxiety and depression. Each item is assigned a score between 0 and 3. There are two final scores, one for anxiety and one for depression, both of which range from 0 to 21 (0–7: normal; 8–10 borderline; 11–21: abnormal). The higher the score, the worse the symptoms.
Pittsburgh Sleep Quality Index (PSQI)	The scale is a 19-item scale that assesses self-perceived sleep quality. Each domain of the scale is assigned a score from 0 to 3. The final score can range from 0 to 21. The higher the score, the worse the sleep quality.

Disability of the Arm, Shoulder and Hand (DASH)	The scale is a 30-item questionnaire that evaluates the disability associated with self-perceived symptoms and function of the upper limbs. Each item is assigned a score from 1 to 5 (5-point Likert scale). The final score ranges from 0 to 100. The higher the score, the more severe the disability.
Reintegration to Normal Living Index (RNLI)	The scale is an 11-item scale that measures the degree to which individuals achieve reintegration into activities of daily living. Each item is assigned a score between 0 and 10. The final score is between 0 and 100. The higher the score, the better the reintegration.
Functional Assessment of Chronic Illness Therapy-Fatigue Scale (FACIT-Fatigue)	The scale is a 13-item scale that assesses the quality of life associated with self-perceived fatigue and its impact on activities of daily living. Each item is assigned a score from 0 to 4 (5-point Likert scale). The final score ranges from 0 to 52. The higher the score, the better the quality of life.
Functional Evaluation of Cancer Therapy-Cognitive Function (FACT-Cog)	The scale is a 37-item scale that evaluates the quality of life associated with deficits in cognitive functions. The scale consists of two subscales measuring perceived cognitive impairments (subscale COG-PCI) and the impact of perceived cognitive impairments on quality of life (subscale Cog-QOL). Each item is assigned a score from 0 to 4 (5-point Likert scale). The final score of the subscale COG-PCI ranges from 0 to 72, while the final score of the subscale COG-QOL ranges from 0 to 16. The higher the score, the better the quality of life.
FACIT Comprehensive Score for Financial Toxicity (FACIT-COST)	The scale is a 12-item scale that evaluates a cancer patient's quality of life associated with self-perceived financial distress. Each item is assigned a score from 0 to 4 (5-point Likert scale). The final score ranges from 0 to 44. The higher the score, the better the quality of life.
Core Set-VR-Onco	This checklist is based on the International Classification of Functioning, Disability and Health (ICF), which was recently adapted and validated to a population of cancer survivors of the Province of Reggio Emilia. The ICF has a hierarchical structure whose different levels are components, chapters, and categories. The four components are Body Functions, Body Structures, Activity and Participation, and Environmental Factors. Each component is divided into chapters, and each chapter comprises several categories. The Core Set-VR-Onco consists of 85 categories: 26 for Body Functions, 33 for Activity and Participation, and 26 for Environmental Factors. For 59 of these categories, it is possible to define whether each represents a problem or not with respect to the return-to-work process (dichotomous answer, problem/no problem); for the remaining 26, it is possible to define whether they influence work or not (dichotomous answer, influences/does not influence). When the answer is affirmative, it is possible to further distinguish the response as a barrier or facilitator (dichotomous answer).
Supportive Care Needs Survey-Short Form (SCNS-SF34)	The scale is a 34-item questionnaire that assesses the perceived needs of cancer patients. The 34 items are divided into five domains. Each item is assigned a score from 1 to 5 (5-point Likert scale), with a total of five final scores, one for each domain: psychological needs (10 items), needs related to the health system and information (11 items), physical and daily living needs (5 items), needs for patient care and support (5 items), and sexual needs (3 items). The final score for each domain ranges from between 0 and 100 (standardized Likert summated score). The higher the score, the more the unmet needs.

Online Resource 3. Recruitment Flow Diagram.

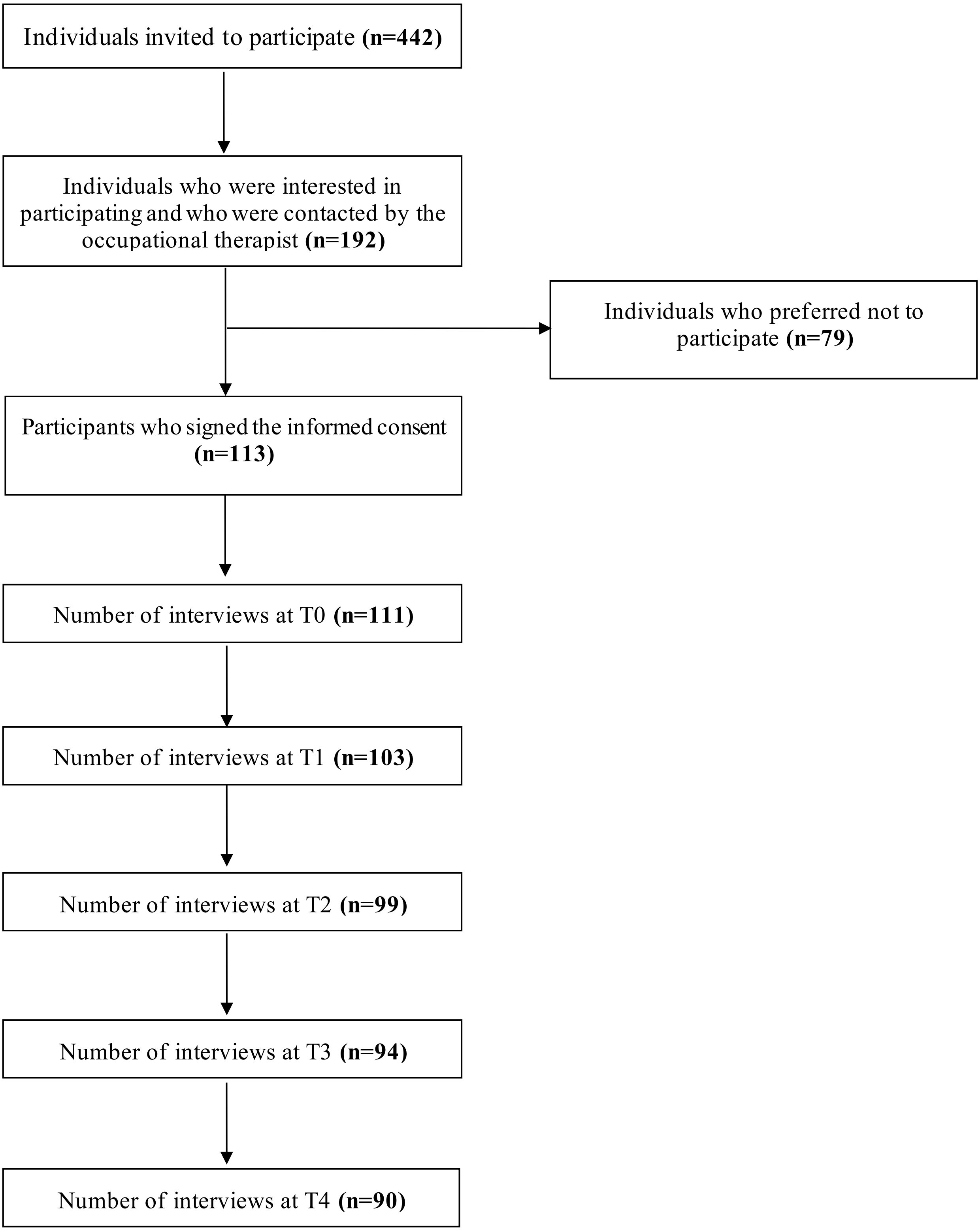

Online Resource 4. Rate of participants who returned to work divided into employees and self-employed.

**Follow-Up Time point**	**Total number of participants whose return to work was recorded**	**Total number of employees**	**n (%) of employees who returned to work**	**Total number of self-employed**	**n (%) of self-employed who returned to work**
T0 baseline	85	76	13 (17.1)	9	5 (55.6)
T1 T0 + 1 month	79	73	31 (42.5)	6	6 (100.0)
T2 T0 + 3 months	76	69	42 (60.9)	6	5 (83.3)
T3 T0 + 6 months	69	62	48 (77.4)	5	4 (80.0)
T4 T0 + 12 months	69	63	58 (92.1)	5	5 (100.0)

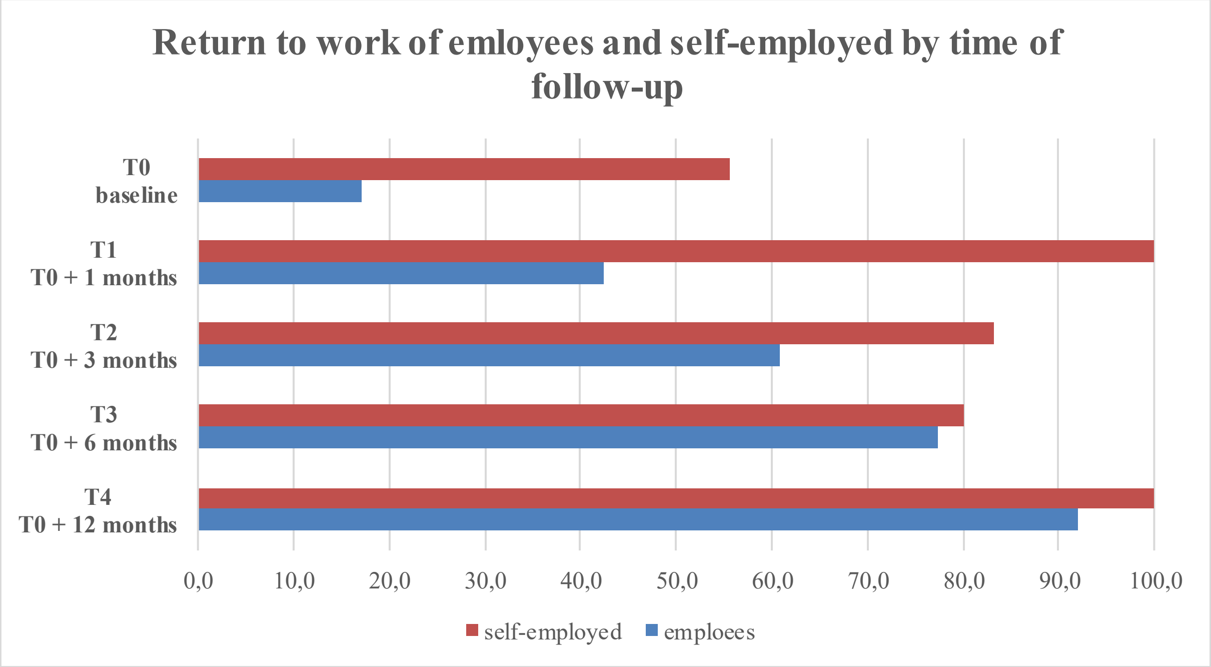

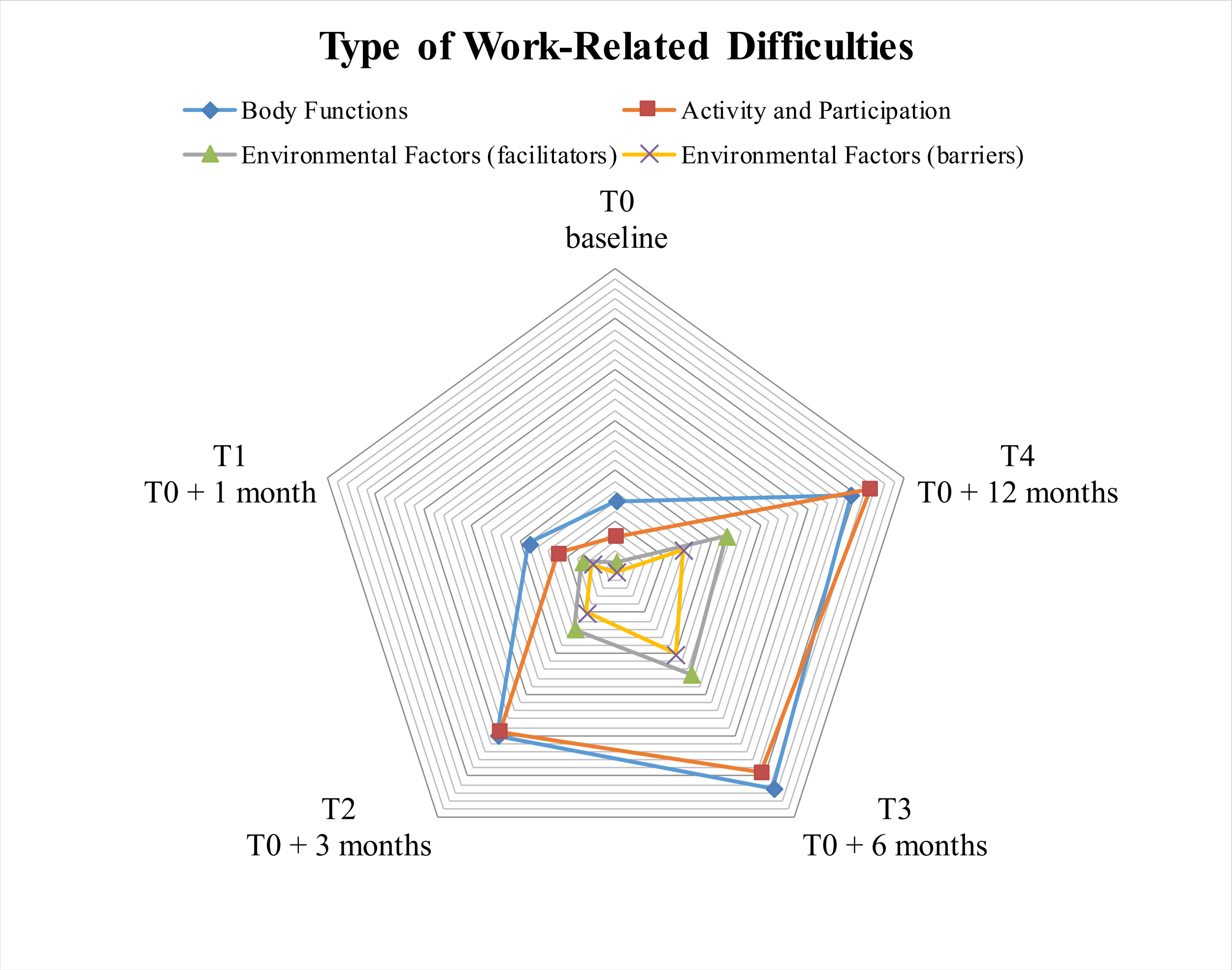

Online Resource 5. Absolute number and type of work-related difficulties reported by each follow-up time point.

Online Resource 6. Description of sick leave.

**Follow-up Time point**	**N. of Participants**	**N. (%) of Participants who Reported Sick Leave**
Diagnosis to surgery	85	45 (52.9)
T0, baseline	85	85 (100.0)
T1, T0 + 1 month	79	64 (81.0)
T2, T0 + 3 months	75	46 (61.3)
T3, T0 + 6 months	67	39 (58.2)
T4, T0 + 12 months	68	40 (58.8)
